# Oxidative Stress-mediated Lipid Peroxidation-derived Lipid Aldehydes in the Pathophysiology of Neurodegenerative Diseases

**DOI:** 10.2174/011570159X342720241014164650

**Published:** 2024-10-21

**Authors:** Kieran Allowitz, Justin Taylor, Kyra Harames, John Yoo, Omar Baloch, Kota V. Ramana

**Affiliations:** 1 Department of Biomedical Sciences, Noorda College of Osteopathic Medicine, Provo, UT-84606, USA

**Keywords:** Oxidative stress, lipid peroxidation, hydroxynonenal, malondialdehyde, Alzheimer’s, Parkinson’s, neurodegenerative disorders

## Abstract

Neurodegenerative diseases such as Alzheimer’s, Parkinson’s, and amyotrophic lateral sclerosis cause damage and gradual loss of neurons affecting the central nervous system. Neurodegenerative diseases are most commonly seen in the ageing process. Ageing causes increased reactive oxygen species and decreased mitochondrial ATP generation, resulting in redox imbalance and oxidative stress. Oxidative stress-generated free radicals cause damage to membrane lipids containing polyunsaturated fatty acids, leading to the formation of toxic lipid aldehyde products such as 4-hydroxynonenal and malondialdehyde. Several studies have shown that lipid peroxidation-derived aldehyde products form adducts with cellular proteins, altering their structure and function. Thus, these lipid aldehydes could act as secondary signaling intermediates, modifying important metabolic pathways, and contributing to the pathophysiology of several human diseases, including neurodegenerative disorders. Additionally, they could serve as biomarkers for disease progression. This narrative review article discusses the biological and clinical significance of oxidative stress-mediated lipid peroxidation-derived lipid aldehydes in the pathophysiology of various neurodegenerative diseases.

## 1 INTRODUCTION

Alzheimer’s disease (AD), Parkinson’s disease (PD), and amyotrophic lateral sclerosis (ALS) are common neurodegenerative disorders that cause neurological and behavioural problems in patients leading to dementia [1-3]. The incidences of these complications are increasing at an alarming rate worldwide [3, 4]. Although ageing seems to be a critical factor associated with the progressive development of neurological conditions, other cellular processes such as protein aggregation, neuronal inflammation, mitochondrial dysfunction, and oxidative stress could play a significant role in causing neurodegenerative diseases [5, 6].

**Fig. (1):**
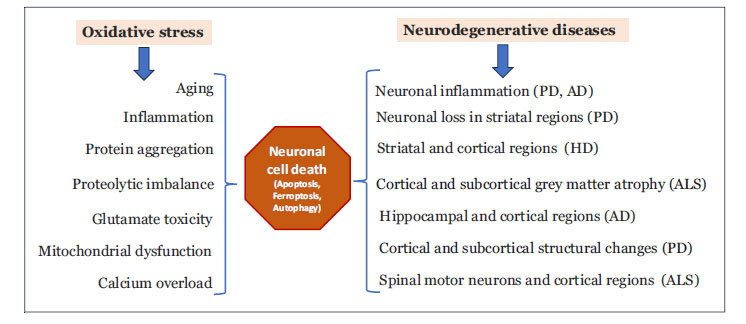
Role of oxidative stress in mediation of neurodegenerative diseases. Increased oxidative stress can cause aging, and aging can also lead to oxidative stress. This leads to impaired protein folding, protein aggregation, altered metabolic pathways, mitochondrial dysfunction, and cellular calcium imbalance. These processes result in neuronal cell death and dysfunction. The initiation of neurodegeneration starts in subcortical regions and spreads across cortical regions as the disease progresses.

Oxidative stress is a major contributing factor to neurological consequences due to ageing [7, 8]. It alters mitochondrial function, protein aggregation, and calcium overload, and increases inflammatory responses, leading to neuronal death and dysfunction (Fig. **1**). The increased production of reactive oxygen species (ROS) causes a disbalance in cellular metabolic pathways, resulting in imbalances in anti- and pro-oxidant pathways and leading to pathological consequences. Several studies have shown that increased free radical formation during ageing and exposure to oxidants leads to damage to proteins and nucleic acids [9, 10]. Furthermore, free radicals attack on cellular membranes, leading to lipid peroxidation, a process that releases several toxic lipid aldehydes such as 4-hydroxy-trans-2-nonenal (HNE) and malondialdehyde (MDA). These lipid peroxidation-derived aldehydes (LDAs) are toxic with the ability to alter several cellular pathways spontaneously by binding to proteins and non-spontaneously activating cellular signaling pathways [11-13]. These lipid aldehydes can post-translationally modify proteins by covalently binding to proteins through interactions with peptide side chains and by forming Michael additions of lipid aldehydes such as HNE to amino acids like Cys, Lys, and His [14, 15]. These modifications could alter the physiological functions of proteins, leading to pathological consequences. Recent studies have shown that these lipid aldehydes are involved in the pathophysiology of neurodegenerative disorders, and indeed, some studies indicate that they could act as biomarkers of Alzheimer’s disease (AD), Parkinson’s disease (PD), Amyotrophic Lateral Sclerosis (ALS), and Huntington’s disease (HD) [16-21].

**Fig. (2):**
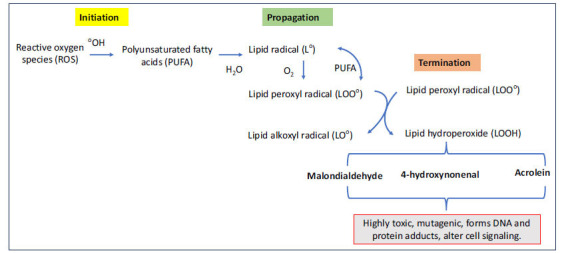
Lipid peroxidation process and generation of lipid aldehyde intermediates. Reactive - free aldehydes target polyunsaturated fatty acids (PUFAs) in membrane phospholipids, initiating lipid peroxidation. This process involves three steps: initiation (ROS attack PUFAs, forming lipid radicals), propagation (lipid radicals react with oxygen, forming lipid peroxyl radicals and unstable lipid peroxides), and termination (lipid radicals combine or are neutralized by antioxidants like vitamin E). Excessive lipid peroxidation disrupts cellular metabolic and signaling pathways, leading to pathological conditions, including neurological diseases.

Reactive free aldehydes’ attack is sensitive to polyunsaturated fatty acids (PUFA) in membrane phospholipids and initiates the lipid peroxidation process [22]. This chain reaction involves several steps (Fig. **2**). The first step is an initiation process, where ROS attack PUFA and abstracts a hydrogen atom from the carbon chain of the fatty acid, giving rise to the formation of a lipid radical. In the next propagation step, the lipid radical reacts with molecular oxygen to form a lipid peroxyl radical, which in turn can attack PUFAs, leading to a chain reaction. During this step, lipid peroxides are formed; they are very unstable and decompose to various reactive products such as HNE and MDA. The last step is the termination process in which the chain reaction is terminated when two lipid radicals combine, or some antioxidants such as vitamin E neutralize lipid radicals required for chain propagation (Fig. **2**). Although lipid peroxidation is a normal physiological process required for some cellular functions, excessive and uncontrolled lipid peroxidation impacts cellular metabolic and signalling pathways leading to various pathological conditions including neurological diseases [22-24]. Thus, the balance between the antioxidant system and oxidative stress is disrupted, favouring in lipid peroxidation and the formation of toxic lipid aldehydes. These lipid aldehydes act as secondary signaling intermediates and alter several cell signaling processes, resulting in disease progression.

Lipid aldehydes, generated as byproducts of oxidative stress-mediated lipid peroxidation, have been implicated in the pathogenesis of neurodegenerative disorders [25-27]. They can induce neuroinflammation, mitochondrial dysfunction, and apoptosis of neuronal cells, resulting in neuronal damage and dysfunction. Apoptotic cell death of neuronal cells is particularly common in neurodegenerative disorders. Furthermore, lipid aldehydes can covalently modify proteins through adduction. Protein-lipid aldehyde adducts can alter the structure and biological function of several important cellular proteins, forming toxic aggregates and inclusion bodies that contribute to neurological complications [28-30]. Additionally, lipid aldehydes can damage mitochondria, and activate inflammatory responses, yielding neuronal inflammation and promoting neuronal degeneration. Some lipid aldehydes can induce DNA damage and genomic instability, leading to mutations that disrupt cellular functions and contribute to the overall pathology of neurodegenerative disorders [31]. Therefore, understanding the contribution of lipid peroxidation-derived aldehydes to neurodegenerative disorders is crucial for developing novel therapeutic strategies aimed at mitigating the effects of oxidative stress. Studies from the last decade or so have shown a significant relationship between LDAs and the pathophysiology of neurodegenerative disorders. In this review article, we discuss recent findings on how LDAs mediate specific neurological complications. We conducted a PubMed search to find articles published in the last 10 years or so, using keywords such as lipid peroxidation, lipid aldehydes, hydroxynonenal, malondialdehyde, acrolein, Alzheimer’s disease, Parkinson’s disease, and neurodegenerative diseases. In addition, we included research articles, comprehensive narrative reviews, meta-analytical studies, systematic reviews, and clinical, and pre-clinical studies. We did not include any studies that show the effects of lipid aldehydes on neurological complications such as migraine, anxiety, multiple sclerosis, and stroke. In this narrative review article, we specifically discuss the significance of these lipid aldehydes in the pathophysiology of various neurodegenerative disorders.

## 2 LIPID ALDEHYDES IN THE PARKINSON’S DISEASE

Parkinson’s Disease (PD) is one of the most common progressive neuromuscular disorders in the world; it affects 0.3% of the general population and about 1% of the population that is 60 years or older [32]. Major motor symptoms include tremors, rigidity, akinesia, and posture and non-motor symptoms include neurobehavioral disorders (*i.e*., depression or anxiety), cognitive decline, and autonomic dysfunction such as orthostatic or hyperhidrosis. These symptoms are caused by the selective deterioration or complete loss of dopaminergic neurons, most notably in the substantia nigra of the midbrain, but also throughout the central, peripheral, and enteric nervous systems. The loss of these neurons is also connected to the manifestation of filamentous aggregations known as Lewy Bodies, which contain misfolded α-Synuclein (αSyn) protein accumulations. While a definitive diagnosis of PD is achievable only *via* autopsy, a clinical diagnosis can be reached by relying heavily on a patient’s history, physical examination, and sustained improvement of symptoms with dopaminergic treatment, such as levodopa [32-34].

The ultimate cause(s) of PD is currently unknown. While approximately 5-10% of PD cases have a genetic foundation, 90-95% of PD cases are idiopathic. Ageing represents the most significant risk factor for PD; it has been observed that the prevalence of PD increases from 1% among those 60 years or older to 3% among those 80 years or older [32]. In the search for the true pathogenic origins of PD, oxidative stress has been postulated and proven throughout the literature as a critical player in the nigral cell death and loss noted in PD [35]. The brain, and more specifically the dopaminergic neurons, are especially susceptible to oxidative stress due to the composition of the brain as well as its high energy demands which require extra amounts of oxygen and mitochondria activity. Few studies indicate that several markers of oxidative stress and DNA damage were increased in PD patients when compared to the control subjects [36-38]. Additionally, glutathione, a crucial cellular antioxidant tripeptide used for the detoxification and reduction of oxidative stress, has been found in decreased levels in the postmortem brain tissue of this patient population when compared to controls [39, 40].

**Fig. (3):**
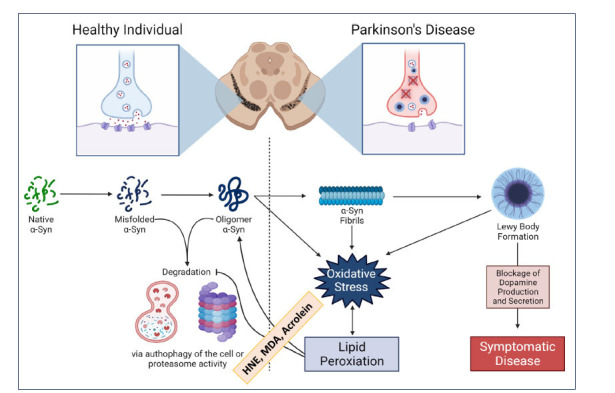
Significance of lipid peroxidation-derived aldehydes in Parkinson’s disease. Lipid peroxidation-derived aldehydes could contribute to oxidative stress-induced pathogenesis of Parkinson’s disease (PD). α-Synuclein (αSyn), a protein involved in synaptic function, accumulates in PD, and is affected by lipid aldehydes. HNE and ONE can modify αSyn, blocking sites necessary for proteasomal degradation, which may lead to further αSyn fibril production and Lewy body formation. This impairment can disrupt dopamine production and signaling, contributing to PD.

Lipid peroxidation is hypothesized to play a role in Parkinson's disease pathogenesis and pathophysiology, given that oxidative stress is known to play a significant role in these processes (Fig. **3**). αSyn is a protein involved in synaptic function and its hallmark accumulation in PD is affected by lipid peroxidation and its byproducts [41, 42]. Considering αSyn's demonstrated affinity for lipid membranes, it seems sensible to propose that there's a good chance αSyn interacts with products of lipid peroxidation [43]. Almandoz-Gil *et al*. demonstrated this idea when they found that HNE and 4-oxo-trans-2-nonenal (4-ONE) modify αSyn covalently *via* Michael addition [44]. When this modification occurs, it blocks necessary sites for proteasomal degradation, which can be the precursor to further αSyn oligomer production and oxidative stress [45].

Animal studies have been crucial to our understanding of PD and the role that oxidative stress and lipid peroxidation play in its pathophysiology. One study focusing on the connection between PD and traumatic brain injury used a rat model to assess their hypothesis concerning acrolein and αSyn accumulation [46]. They discovered that the increased levels of acrolein found in rats after blast-induced traumatic brain injury were accompanied by increased modification and oligomerization αSyn, indicating that acrolein plays a role in the pathophysiology of PD. In other studies, utilizing *in vitro* and *in vivo* techniques, HNE was discovered to not only react with αSyn to promote αSyn aggregation and oligomerization but also induce the release of pathogenic αSyn with greater toxicity [47, 48]. These studies also demonstrated that HNE interferes with the process of proteasomal degradation and cell autophagy, inhibiting the body’s natural process of prion removal.

Results from human research have shown similar findings to those of animal and *in vitro* studies. In two separate studies, elevated levels of lipid aldehydes MDA, HNE, and ONE were discovered in both the plasma and CSF of living patients diagnosed with PD [49, 50]. As only a definite diagnosis can be made *via* autopsy, it is crucial to have data from postmortem evaluations to confirm this connection between PD and lipid peroxidation. In their studies, Yoritaka *et al*. discovered elevated MDA and HNE adducts in the brain tissues of postmortem PD patients and that these adducts were selectively found in substantia nigra tissue, suggesting a connection specifically to PD [51]. Another postmortem study by Castellami *et al*. found HNE adducts in Lewy bodies of PD patients’ brain stems and cortexes [52].

Further exploration of lipid peroxidation in PD is valuable not only in improving our understanding of the pathophysiology of the disease but also in helping medical professionals better take care of their patients. Currently, there are no available pharmacological approaches to the suppression of neural loss seen in PD. Subsequently, treatment is presently restricted to symptom management, most often *via* dopamine treatment. However, in recent years, studies have postulated that a better understanding of the presence and contribution of lipid peroxidation to PD may lead to novel therapies that not only improve symptom management but also better control of the disease itself. For example, the modification of regulators of G protein signaling (RGSs), specifically RGS4, has been connected to the pathology of PD. In a study by Monroy *et al*., HNE was found to specifically inhibit RGS4 activity [53]. This study suggested that focusing on the interaction between HNE and RGS4 could help in potential nondopaminergic treatments for PD patients. In another study, the use of aldehyde scavengers, more specifically the drug dimercaprol, was demonstrated to have a neuroprotective effect *in vitro* as well as in animal models of PD [54]. These results not only elucidate the acrolein-mediated pathogenesis of PD but also provide a robust case for PD treatment options that focus on decreasing lipid aldehydes. Treatments like this appear to have great promise, however, this remains an area of research that necessitates further exploration.

## 3 LIPID ALDEHYDES IN THE HUNTINGTON’S DISEASE

Huntington’s disease (HD) is a genetic neurodegenerative disease that leads to the breakdown of nerves in the brain. The disorder is caused by polyglutamine or cytosine-adenosine-guanine (CAG) repeats in the Huntingtin protein [55-57]. Huntingtin (HTT), located on the short arm of chromosome 4, is found in many tissues, but the highest levels of the protein are found in the testes and brain. More specifically, HTT levels are centralized in the glial cells as well as all neurons. The Huntingtin protein is involved in many functions such as chemical signaling, binding proteins, and preventing apoptosis [51].

**Fig. (4):**
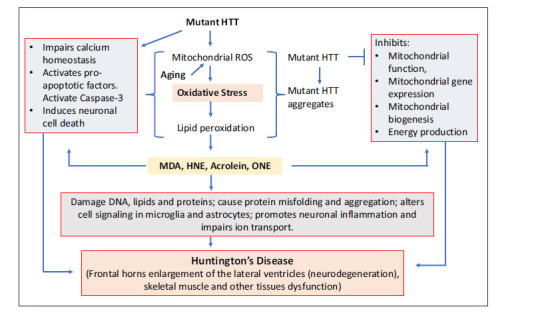
Role of lipid peroxidation-derived aldehydes in Huntington’s disease: The mutation in the HTT gene leads to the production of toxic mutated huntingtin protein aggregates, which disrupt various cellular processes and contribute to neuronal dysfunction and death. Oxidative stress-induced lipid peroxidation-derived aldehydes play a critical role in this process by causing mitochondrial dysfunction, impairing antioxidant defenses, increasing neuronal cell death, causing DNA and protein damage, and inducing neuronal inflammation, all of which accelerate the progression of Huntington's disease.

Mutations causing excessive polyglutamine expansions within HTT impair the function of the protein, leading to neurological symptoms associated with Huntington’s disease (Fig. **4**). Those with HD may experience many symptoms, including difficulty with speech or swallowing, involuntary jerking (chorea), impaired gait, and other motor abnormalities [55, 56]. Skeletal muscular atrophy, weight loss, and autonomic disruptions are a few other clinical signs. Although HD first presents as psychiatric symptoms, the most common being depression and other mood disorders. Other symptoms, such as motor abnormalities and dementia, develop over the next 15-20 years. HD typically manifests in patients who are 30 to 50 years old. Any symptoms before the age of 20 are considered juvenile Huntington’s disease [57].

In HD, elevated levels of oxidative stress-generated free radicals and lipid aldehydes have been detected [58]. Several studies suggest the involvement of mitochondria function and oxidative stress in the pathophysiology of HD [59-61]. Lipid peroxidation products such as HNE, MDA, and acrolein may affect Huntington's protein and other related proteins involved in HD [62-64].

Recent studies involving rodent models of Huntington’s disease have suggested that HD may also be initiated by protein aggregation composed of fragmented mutant HTT. Elevated levels of MDA were found in areas of degeneration within the HD-affected brain, although it is not known whether high levels of MDA serve as the product of oxidative stress or as a causative agent [65, 66]. It is also suspected that HD cell death pathogenesis may be related to a gain of function mutation of HTT and that the mutation may be a result of oxidative damage. HD has been shown to exhibit a specific pattern of cell damage in the brain, affecting selective neurotransmitter pathways caused by HTT.

Antioxidants' effect on oxidative stress and HD were studied using acrolein as a marker for oxidative stress. Antioxidants were shown to be effective in slowing the progression of HD in transgenic mouse models. Lower levels of acrolein in HD brain were also exhibited [65-70]. These studies suggest the potential of oxidative stress in the pathophysiology of HD and its suppression by several antioxidants. A meta-analysis study by Tang *et al.* suggests that lipid aldehyde biomarkers were increased in the blood of HD patients [71].

Huntington’s disease has been widely studied, and a major culprit of HD has been narrowed down to the huntingtin protein. It is generally accepted that HTT dysfunction is due to polyglutamine expansions. However, recent studies have shown that HTT aggregates or gain of function mutations may also contribute to the pathogenesis of HD [72-74].

## 4 LIPID ALDEHYDES IN THE ALZHEIMER’S DISEASE

Alzheimer’s disease (AD), a well-known neurodegenerative disorder prevalent in older individuals, is characterized by a progressive cognitive decline leading to dementia [75-77]. While the exact cause remains uncertain, extensive research has identified risk factors such as age, family history, and genetics [77]. Understanding the pathophysiology of AD has become essential, as cases are expected to climb to 16 million in the United States by 2050.

Histologic hallmarks of Alzheimer’s include amyloid plaques, neurofibrillary tangles, and synapse loss. To explain the disease's pathogenesis, several studies found the involvement of oxidative stress, redox imbalance, and neuronal cell damage [77-80]. LDAs have been shown to mediate oxidative damage leading to AD. Mark *et al*. [81] have demonstrated that HNE induces oxidative damage by promoting free radical production in amyloid beta-peptide (Aβ). Primary hippocampal cell cultures quantified HNE to show its role in disrupting neuronal ion homeostasis and causing cell death, offering insights into Alzheimer’s complex pathogenesis. Excessive consumption of deep-fried foods, especially those cooked in ω-6 PUFA-rich vegetable oils, is linked to lifestyle diseases like Alzheimer's, type 2 diabetes, and obesity, potentially due to the lipid peroxidation product HNE. These lipid aldehydes could impair the protective chaperone protein Hsp70.1, disrupting protein recycling and lysosomal stability, cell degeneration, and death across various neuronal tissues and other tissues, leading to neurological and metabolic diseases [82, 83]. A recent study by Yamshima *et al*. [84] has indicated that aldehyde dehydrogenase 2 (ALDH2) detoxifies HNE, which is implicated in ischemic neuronal death *via* the calpain-cathepsin cascade and in AD. They have also suggested that HNE-induced Hsp70.1 disorder, not amyloid β, is central to AD pathology.

The involvement of lipid aldehydes in AD is further supported by the brain's vulnerability to oxidative stress due to high oxygen utilization, redox metal ions, and poor antioxidant systems. The brain is susceptible to lipid peroxidation, forming various aldehydes, including HNE, malondialdehyde, and acrolein. Increased levels of these aldehydes and their adducts have been observed in AD brains. Elevated F2-isoprostanes, neuroprostanes, and lipid aldehydes in brain regions are associated with high oxidative stress, suggesting that oxidative damage is significant even in the initial stages of AD, including mild cognitive impairment (MCI) and preclinical phases [85-87]. These reactive LDAs bind *via* Michael in addition to specific amino acids, altering their function and conferring neurotoxicity. Butterfield *et al*. [88] observed in transgenic mice modelling Alzheimer’s that the Met35 residue of A beta peptide was responsible for oxidative stress, linking methionine oxidation to lipid peroxidation-induced aldehydes in neurodegenerative diseases like AD.

**Fig. (5):**
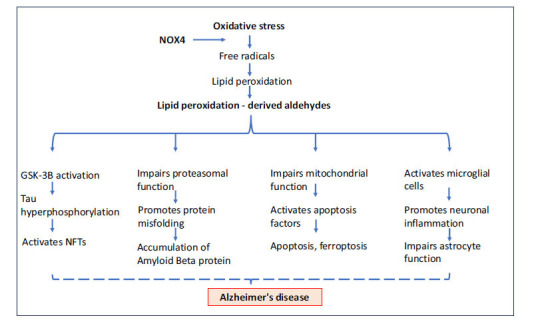
Significance of lipid aldehydes in the pathology of Alzheimer’s disease. Increased oxidative stress during aging and elevated NOX4 levels further induce the peroxidation of membrane lipids, forming lipid aldehyde intermediates. These aldehydes could activate GSK-3B by inhibiting PP2A, leading to Tau hyperphosphorylation. They are also responsible for the accumulation of amyloid beta protein by interrupting proteasomal function, causing neuronal cell death by activating pro-apoptotic factors such as caspase 3. Additionally, they contribute to increased neuronal inflammation and astrocyte cytotoxicity, ultimately leading to Alzheimer’s disease.

Neurodegenerative diseases are characterized by inflammation and oxidative stress-induced neuronal damage. NADPH oxidase 4 (NOX4) plays a key role in producing reactive oxygen species and initiating the inflammatory response. NOX4 is involved in activating glial cells and disrupting neuronal functions, suggesting that targeting NOX4 in astrocytes could reduce oxidative damage and neuroinflammation [89-91]. Park *et al*. [92] have explored the impact of NOX4 on astrocytes in AD patients. Extracted impaired cells from the cerebral cortex showed elevated levels of HNE and malondialdehyde due to NOX4 overexpression, further linking Alzheimer’s to oxidative damage induced by lipid peroxidation-derived aldehydes (Fig. **5**). Increased fasting glucose and decreased HDL cholesterol in AD patients indicate metabolic abnormalities progressing from hyperglycemia to AD. A study by Sanotra *et al*. [93] has found that HNE adducts serve as better diagnostic markers than Aβ for both conditions. They have shown increased serum HNE-Aβ peptide levels and decreased responding autoantibodies, particularly IgM, in hyperglycemic AD groups, suggesting that immunity disturbances contribute to AD pathogenesis. The same group of researchers have also indicated that increased levels of acrolein adducts found in AD were correlated with metabolic syndrome conditions [94]. They found that in human samples, acrolein adducts were significantly higher in the AD-M group, with a notable reduction in anti-acrolein-Aβ autoantibodies, particularly IgM, suggesting that the depletion of these antibodies during the progression from metabolic syndrome to AD might contribute to disease development.

Further, HNE-protein Michael adducts, and glutathione-HNE adducts are increased in the AD hippocampus and other brain regions, contributing to oxidative stress [95]. The altered activity of enzymes like aldehyde dehydrogenases, NOX, and proteasome in AD brains exacerbates lipid peroxidation and accumulation of cytotoxic biomolecules. Additionally, MDA levels are significantly increased and colocalized with neuropathological markers in AD brains. Improved CSF drainage and antioxidant treatments have shown promise in reducing lipid peroxidation in AD, offering potential avenues for disease management. However, conflicting findings regarding specific HNE adducts and their levels in AD highlight the complexity of lipid peroxidation processes in the disease. Even though some research aims to mimic specific clinical characteristics, such the buildup of amyloid plaque, it is unable to fully capture the comprehensive symptoms that characterize Alzheimer's disease, such as neuronal loss and neurofibrillary tangles that are controlled by lipid aldehydes. This complexity in pathophysiology has made treatment management challenging. Drugs like donepezil, galantamine, rivastigmine, and memantine alleviate symptoms but do not cure the underlying disease. Further research is needed to elucidate the precise mechanisms and develop targeted interventions against lipid peroxidation-induced damage in AD.

## 5 LIPID ALDEHYDES IN THE AMYOTROPHIC LATERAL SCLEROSIS

Amyotrophic Lateral Sclerosis (ALS) is a progressive neurodegenerative disorder characterized by the gradual degeneration of motor neurons in the brain and spinal cord. Its estimated annual incidence is 1 to 2 cases per 100,000 individuals globally. While ALS could affect people of any age, it most commonly manifests in individuals between the age range of 40-70. The major symptoms included in this degeneration are muscle weakness, spasticity, and eventually paralysis as voluntary muscle control is lost [96, 97]. The exact cause of ALS is not fully understood, though a combination of genetic and environmental factors is believed to contribute to its development. Recent studies also suggest that oxidative stress is another causative factor for developing ALS [98-100]. Hemerková and Valis have suggested that antioxidant enzymes and appropriate antioxidant protection played a role in several ALS cases [98]. Notably, the mutation of superoxide dismutase (SOD1) was identified in 20% of familial ALS cases, underlining the importance of these antioxidant mechanisms in mitigating the effects of oxidative stress. From an alternative perspective, Carrera-Julia *et al*. [101] have suggested a potential role of NAD^+^ levels in reducing mitochondrial oxidative stress. Lower bioavailability of NAD^+^ was consistently found in several neurodegenerative diseases, indicating that NAD^+^/NADH homeostasis plays a crucial role in neuronal function. Further, a Mediterranean diet containing antioxidants nicotinamide riboside and pterostilbene or a Mediterranean diet containing coconut oil improves anthropometric parameters in ALS patients [102]. A recent study by the same group indicated nutritional and dietary changes affect ALS in Spanish patients [103].

**Fig. (6):**
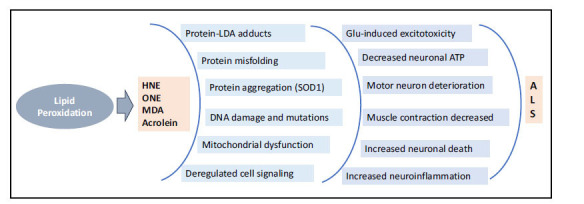
Contribution of oxidative stress-induced lipid aldehydes in the pathogenesis of Amyotrophic lateral sclerosis. Oxidative stress-induced lipid aldehydes significantly contribute to the pathogenesis of Amyotrophic Lateral Sclerosis (ALS). These aldehydes are implicated in the formation of protein aggregates, DNA damage, disruption of mitochondrial functions, and alteration of cell signaling. These processes lead to increased inflammation, progressive degeneration of motor neurons, and decreased muscle contraction. These processes collectively drive the progression of ALS.

There is also a compelling indication that lipid aldehydes derived from peroxidation processes play a pivotal role in the pathogenesis of ALS [104-108]. Indeed, some studies indicate that altered lipid metabolism in the spinal cord could contribute to ALS [109-111]. Further, Phan *et al*. have indicated increased lipid aldehyde MDA in ALS serum [112]. These studies, thus, suggest that lipid aldehydes such as HNE could act as biomarkers of ALS progression (Fig. **6**).

## 6 LIPID ALDEHYDES IN NEURODEGENERATIVE ATAXIAS

Less understood, yet showing increasing merit, are the effects of lipid peroxidation secondary products and the roles they play in the pathophysiology of neurodegenerative ataxias. Ataxias are a group of diseases generally identified by a lack of coordination and control over voluntary movements, affecting balance, speech, and motor skills [113-115]. They can result from damage to the nervous system, particularly the cerebellum, due to genetic conditions, acquired causes like stroke, alcohol abuse, and unknown etiological factors [116-118]. Epidemiologically, ataxias are relatively rare, with hereditary ataxias such as Friedreich ataxia affecting about 1 in 50,000 Caucasians, while acquired ataxias vary in prevalence depending on the underlying cause [119]. Many forms of ataxia are related to neurodegenerative diseases, where progressive damage to the nervous system, especially the cerebellum, leads to worsening symptoms over time, often requiring long-term management. In addition to acute cerebellar injury, ataxia may also precipitate from deficiencies of vitamin B1 and B12 [120, 121]. The major ataxias include Friedreich’s ataxia which is due to mutations in the FXN gene causing mitochondrial dysfunction and increased oxidative stress. Spinocerebellar ataxias are also a group of hereditary diseases due to mutations in multiple genes. Similarly, Ataxia-telangiectasia is due to mutations in the ATM gene that alter the immune system and increase the risk of developing cancers [122, 123]. Cerebellar ataxia is due to multiple system atrophy and causes significant neurodegeneration in the cerebellum and other brain regions. Several studies demonstrate that oxidative stress plays a significant role in the development of various types of neurodegenerative ataxias [124-126]. In ataxias, oxidative stress could lead to the deterioration of neurons, particularly in the cerebellum, which is crucial for motor coordination and other symptoms of ataxia, such as impaired balance, coordination, and motor skills. In addition, in Friedreich's ataxia, oxidative stress leads to increased ROS production that affects mitochondrial function and subsequent neuronal damage [127-129]. The free radical-mediated oxidative damage is detrimental in ataxia due to the high metabolic demands and vulnerability of cerebellar neurons.

Lipid peroxidation is also a significant process in the pathology of ataxia, contributing to neuronal damage and disease progression. In ataxia, particularly in conditions like Friedreich's ataxia and spinocerebellar ataxias, increased lipid peroxidation is observed, highlighting the importance of oxidative damage in these disorders [130, 131]. HNE has been noted as more toxic yet less stable than MDA, resulting in apoptosis at low concentrations and necrosis when high concentrations [132]. This cytotoxic effect likely contributes to the physical causes of ataxia *via* neural degeneration. Ataxia such as cerebellar and Friedreich ataxia, is characterized by mitochondrial dysfunction resulting from iron accumulation leading to oxidative stress and damage-induced degeneration of nervous system tissues [133, 134]. Aldehydic lipid peroxidation products play a role here, similar to previously mentioned neurological disorders, in that these reactive aldehydes contribute to mitochondrial dysfunction and impair the mitigation of oxidative stress. Another genetically linked ataxia is Ataxia-telangiectasia, where mutations accumulate due to defects in double-stranded DNA repair mechanisms and are associated with immunodeficiency. While the specificity of secondary lipoxidation products for Ataxia Telangiectasia Mutated Protein Kinase (ATM) is poorly understood, it should still be considered vulnerable to adduct formation by MDA and HNE [135]. ATM serves roles in autophagy and mitigation of oxidative stress; modification of ATM by secondary lipoxidation products could result in the accumulation of protein aggregates and increased oxidative stress [136]. A few studies indicate the accumulation of lipid aldehydes such as HNE during ataxia. Maciejczyk *et al*. [137] have shown increased oxidative damage, HNE, and 8-isoprostane levels in children with ataxia telangiectasia. Additional studies are required to understand the critical role of lipid peroxidation-derived aldehydes in the pathology of ataxia. Similarly, Andrade *et al.* [138] have shown increased MDA along with other oxidative biomarkers in ataxia telangiectasia patients.

## CONCLUSION AND FUTURE PERSPECTIVES

Although several studies indicate a definitive role of LDAs in the progression of neurodegenerative diseases, their exact role as a cause or consequence of neurodegenerative diseases is not yet clear. Zhang *et al*. [139] have indicated that HNE is associated with PD by increasing the αSyn aggregation and neuronal loss. Similarly, some studies have also indicated that HNE causes αSyn oligomerization [44, 140, 141]. In contrast, αSyn in association with ferrous ions could generate oxidative stress in the neurons which could increase LDA formation and interact with mitochondrial proteins [142-144]. Similarly, several other studies have also shown that HNE and MDA are early biomarkers of AD and PD, indicating that they are involved in the progression of neurodegenerative diseases [145, 146]. Based on the current evidence, LDAs could exacerbate neurodegenerative diseases by damaging cellular components such as proteins, nucleic acids, and lipids, leading to damage to neuronal tissue. However, LDAs may not be the potential initial triggering factors of these diseases. Most neurodegenerative diseases may be due to gene mutations, environmental and lifestyle changes, ageing, and other biochemical abnormalities that lead to oxidative stress-mediated lipid peroxidation may play a contributing role. Thus, while lipid peroxidation-derived aldehydes could play a critical role in the progression of neurodegenerative diseases, they might not be primarily responsible for underlying pathophysiology.

Therefore, a deeper understanding of the precise molecular mechanisms by which these compounds contribute to neuronal damage and initiate or promote neurodegeneration is essential. This includes exploring their interactions with proteins, DNA, and cellular membranes, and how these interactions lead to the formation of neurofibrillary tangles and other pathological features of diseases such as AD and PD. Understanding these mechanisms not only provides valuable insights into the molecular basis of neurological diseases but also offers potential avenues for therapeutic interventions. Further, the accumulation of LDAs is a common feature seen in many neurodegenerative diseases, the differences between these diseases arise from various unique underlying mechanisms, pathways, and nature of affected brain regions. As a secondary effect, LDAs could impair cellular functions, promoting the damage initiated by other primary mechanisms. For example, it has been shown that in AD, amyloid-β plaques and tau tangles serve as primary contributors. Similarly, in PD, the accumulation of αSyn and the damage of dopaminergic neurons play a critical role. The different forms of LDA interactions with cellular proteins and their specific susceptibility to different neurons could also contribute to the variability in the disease processes. Although LDAs could be essential factors in neurodegenerative diseases, their involvement in the mechanisms, such as protein aggregation, mitochondrial dysfunction, and genetic mutations, often play a significant role in identifying the different characteristics of each neurodegenerative disease [145, 146].

Therefore, extensive research in this field is crucial to unravel the intricate connections between LDAs and their involvement in neurological disorders, paving the way for innovative treatments aimed at mitigating oxidative stress and improving patient outcomes. HNE and HNE adducts, by activating NF-κB-mediated pro-inflammatory responses and disrupting Nrf2-mediated anti-oxidative responses, could cause neuronal cell damage, neuronal inflammation, and apoptosis. Mitochondrial dysfunction, a consequence of increased ROS production, further complexes the neurodegenerative processes observed in several neurological disorders. The specific patterns of cell damage in the brain affecting neurotransmitter pathways highlight the intricate relationship between oxidative stress and selective neuronal vulnerability in these complications. Therefore, reactive lipid aldehydes could be viable targets for clinical therapies aimed at delaying neurological diseases.

Recent studies indicate that antioxidants emerge as potential therapeutic interventions to mitigate the effects of oxidative stress-related diseases, including neurodegenerative disorders [147-149]. Indeed, plant-based antioxidants such as curcumin, green tea, quercetin, and resveratrol, as well as vitamins such as A, C, D and E, have been shown to have the potential to mitigate LDA toxicity and provide neuroprotection [150-153]. Further, pre-clinical studies indicate that these antioxidants can reduce oxidative stress, decrease amyloid-β production, and improve cognitive and motor functions in cell culture and animal models of neurodegenerative diseases. These studies suggest that LDAs are central to the pathophysiology of neurodegenerative diseases and might indicate that neutralizing LDAs with antioxidants could be a therapeutic approach. However, the limited success of antioxidant therapies in clinical trials suggests that LDAs alone may not play a key role in the disease progression [154]. Instead, these aldehydes may be associated with other pathological processes, such as protein aggregation, mitochondrial dysfunction, and inflammation, which might collectively contribute to neurodegenerative diseases. This demonstrates that targeting LDAs alone may not be insufficient to control the disease process and more comprehensive approaches addressing the multiple interlinked cellular mechanisms still need to be investigated.

In conclusion, the interplay between genetic mutations, oxidative stress, and protein dysfunction underscores the multifaceted nature of neurogenerative diseases. Controlling the prevalence of neurodegenerative disorders requires ongoing research and clinical efforts focused on reactive lipid aldehydes that combine basic science, biomarker development, innovative therapeutic approaches, and preventive strategies to mitigate their detrimental effects on neuronal health.

## LIST OF ABBREVIATIONS

Aβ: Amyloid Beta-peptide
AD: Alzheimer’s Disease
ALDH: Aldehyde Dehydrogenase
ALS: Amyotrophic Lateral Sclerosis
αSyn: α-synuclein
GSH: Glutathione
GST: Glutathione-S-transferase
HD: Huntington’s Disease
HNE: 4-hydroxynonenal
HSP: Heat Shock Protein
HTT: Huntington
IgM: Immunoglobulin M
LDAs: Lipid Peroxidation-derived Aldehydes
MDA: Malondialdehyde
NAD: Nicotinamide Adenine Dinucleotide
NDs: Neurological Disorders
NF-κB: Nuclear Factor Kappa B Binding Protein
NFTs: Neurofibrillary Tangles
NOX: NADPH Oxidase
Nrf2: Nuclear Factor-erythroid Factor 2-related Factor 2
ONE: 4-oxo-trans-2-nonenal
PD: Parkinson’s Disease
PP2A: Protein Phosphatase 2A
PUFA: Polyunsaturated Fatty Acids
ROS: Reactive Oxygen Species
SOD: Superoxide Dismutase

## References

[1] Adamu A., Li S., Gao F., Xue G. (2024). The role of neuroinflammation in neurodegenerative diseases: Current understanding and future therapeutic targets.. Front. Aging Neurosci..

[2] Lamptey R.N.L., Chaulagain B., Trivedi R., Gothwal A., Layek B., Singh J. (2022). A review of the common neurodegenerative disorders: Current therapeutic approaches and the potential role of nanotherapeutics.. Int. J. Mol. Sci..

[3] Wilson D.M., Cookson M.R., Van Den Bosch L., Zetterberg H., Holtzman D.M., Dewachter I. (2023). Hallmarks of neurodegenerative diseases.. Cell.

[4] Van Schependom J., D’haeseleer M. (2023). Advances in neurodegenerative diseases.. J. Clin. Med..

[5] Cheslow L., Snook A.E., Waldman S.A. (2024). Biomarkers for managing neurodegenerative diseases.. Biomolecules.

[6] Chand Dakal T., Choudhary K., Tiwari I., Yadav V., Kumar Maurya P., Sharma N.K. (2024). Unraveling the triad: Hypoxia, oxidative stress and inflammation in neurodegenerative disorders.. Neuroscience.

[7] Olufunmilayo E.O., Gerke-Duncan M.B., Holsinger R.M.D. (2023). Oxidative stress and antioxidants in neurodegenerative disorders.. Antioxidants.

[8] Li J.O.W., Li W., Jiang Z.G., Ghanbari H. (2013). Oxidative stress and neurodegenerative disorders.. Int. J. Mol. Sci..

[9] Butterfield D.A., Boyd-Kimball D. (2019). Redox proteomics and amyloid β-peptide: Insights into Alzheimer disease.. J. Neurochem..

[10] Butterfield D.A., Perluigi M. (2017). Redox proteomics: A key tool for new insights into protein modification with relevance to disease.. Antioxid. Redox Signal..

[11] Sonowal H., Ramana K.V. (2019). 4-hydroxy-trans-2-nonenal in the regulation of anti-oxidative and pro-inflammatory signaling pathways.. Oxid. Med. Cell. Longev..

[12] Shoeb M., Ansari N., Srivastava S., Ramana K. (2013). 4-hydroxynonenal in the pathogenesis and progression of human diseases.. Curr. Med. Chem..

[13] Li Y., Zhao T., Li J., Xia M., Li Y., Wang X., Liu C., Zheng T., Chen R., Kan D., Xie Y., Song J., Feng Y., Yu T., Sun P. (2022). Oxidative stress and 4-hydroxy-2-nonenal (4-HNE): Implications in the pathogenesis and treatment of aging-related diseases.. J. Immunol. Res..

[14] Butterfield D.A., Mattson M.P. (2020). Apolipoprotein E and oxidative stress in brain with relevance to Alzheimer’s disease.. Neurobiol. Dis..

[15] Spickett C.M., Pitt A.R. (2020). Modification of proteins by reactive lipid oxidation products and biochemical effects of lipoxidation.. Essays Biochem..

[16] Cioffi F., Adam R.H.I., Bansal R., Broersen K. (2021). A review of oxidative stress products and related genes in early Alzheimer’s disease.. J. Alzheimers Dis..

[17] Abeer M.I., Abdulhasan A., Haguar Z., Narayanaswami V. (2023). Isoform-specific modification of apolipoprotein E by 4-hydroxynonenal: Protective role of apolipoprotein E3 against oxidative species.. FEBS J..

[18] Jaganjac M., Milkovic L., Gegotek A., Cindric M., Zarkovic K., Skrzydlewska E., Zarkovic N. (2020). The relevance of pathophysiological alterations in redox signaling of 4-hydroxynonenal for pharmacological therapies of major stress-associated diseases.. Free Radic. Biol. Med..

[19] Arnett D., Quillin A., Geldenhuys W.J., Menze M.A., Konkle M. (2019). 4-hydroxynonenal and 4-oxononenal differentially bind to the redox sensor MitoNEET.. Chem. Res. Toxicol..

[20] Kabuta C., Kono K., Wada K., Kabuta T. (2015). 4-hydroxynonenal induces persistent insolubilization of TDP-43 and alters its intracellular localization.. Biochem. Biophys. Res. Commun..

[21] Disatnik M.H., Joshi A.U., Saw N.L., Shamloo M., Leavitt B.R., Qi X., Mochly-Rosen D. (2016). Potential biomarkers to follow the progression and treatment response of Huntington’s disease.. J. Exp. Med..

[22] Ayala A., Muñoz M.F., Argüelles S. (2014). Lipid peroxidation: Production, metabolism, and signaling mechanisms of malondialdehyde and 4-hydroxy-2-nonenal.. Oxid. Med. Cell. Longev..

[23] Zarkovic K., Jakovcevic A., Zarkovic N. (2017). Contribution of the HNE-immunohistochemistry to modern pathological concepts of major human diseases.. Free Radic. Biol. Med..

[24] Zhang H., Forman H.J. (2017). 4-hydroxynonenal-mediated signaling and aging.. Free Radic. Biol. Med..

[25] Adibhatla R.M., Hatcher J.F. (2008). Altered lipid metabolism in brain injury and disorders.. Subcell. Biochem..

[26] Di Domenico F., Tramutola A., Butterfield D.A. (2017). Role of 4-hydroxy-2-nonenal (HNE) in the pathogenesis of Alzheimer disease and other selected age-related neurodegenerative disorders.. Free Radic. Biol. Med..

[27] Sultana R., Perluigi M., Butterfield D.A. (2013). Lipid peroxidation triggers neurodegeneration: A redox proteomics view into the Alzheimer disease brain.. Free Radic. Biol. Med..

[28] Žarković N., Gęgotek A., Łuczaj W., Jaganjac M., Šunjić S.B., Žarković K., Skrzydlewska E. (2024). Overview of the lipid peroxidation measurements in patients by the enzyme-linked immunosorbent assay specific for the 4-hydroxynonenal-protein adducts (4-HNE-ELISA).. Front. Biosci. . (Landmark Ed),.

[29] Barrera G., Pizzimenti S., Ciamporcero E.S., Daga M., Ullio C., Arcaro A., Cetrangolo G.P., Ferretti C., Dianzani C., Lepore A., Gentile F. (2015). Role of 4-hydroxynonenal-protein adducts in human diseases.. Antioxid. Redox Signal..

[30] Milkovic L., Zarkovic N., Marusic Z., Zarkovic K., Jaganjac M. (2023). The 4-hydroxynonenal-protein adducts and their biological relevance: Are some proteins preferred targets?. Antioxidants.

[31] Camporez D., Belcavello L., Almeida J.F.F., Silva-Sena G.G., Pimassoni L.H.S., Morelato R.L., do Carmo Pimentel Batitucci M., de Paula F. (2021). Positive association of a Sirt1 variant and parameters of oxidative stress on Alzheimer’s disease.. Neurol. Sci..

[32] Deliz J.R., Tanner C.M., Gonzalez-Latapi P. (2024). Epidemiology of Parkinson’s disease: An update.. Curr. Neurol. Neurosci. Rep..

[33] Postuma R.B., Berg D., Stern M., Poewe W., Olanow C.W., Oertel W., Obeso J., Marek K., Litvan I., Lang A.E., Halliday G., Goetz C.G., Gasser T., Dubois B., Chan P., Bloem B.R., Adler C.H., Deuschl G. (2015). MDS clinical diagnostic criteria for Parkinson’s disease.. Mov. Disord..

[34] Nabizadeh F., Seyedmirzaei H., Rafiei N., Maryam Vafaei S., Shekouh D., Mehrtabar E., Mirzaaghazadeh E., Mirzaasgari Z. (2024). Global prevalence and incidence of young onset Parkinson’s disease: A systematic review and meta-analysis.. J. Clin. Neurosci..

[35] Cai P., Wang J., Xu J., Zhang M., Yin X., He S., Zhuang J. (2024). V-set and immunoglobulin domain containing 4 inhibits oxidative stress, mitochondrial dysfunction, and inflammation to attenuate Parkinson’s disease progression by activating the JAK2/STAT3 pathway.. J. Neuroimmunol..

[36] Pfeifer G.P. (2024). DNA damage and Parkinson’s disease.. Int. J. Mol. Sci..

[37] Sadeghian Z., Eyvari-Brooshghalan S., Sabahi M., Nourouzi N., Haddadi R. (2022). Post treatment with Gastrodin suppresses oxidative stress and attenuates motor disorders following 6-OHDA induced Parkinson disease.. Neurosci. Lett..

[38] Zhang J., Perry G., Smith M.A., Robertson D., Olson S.J., Graham D.G., Montine T.J. (1999). Parkinson’s disease is associated with oxidative damage to cytoplasmic DNA and RNA in substantia nigra neurons.. Am. J. Pathol..

[39] Shukla D., Goel A., Mandal P.K., Joon S., Punjabi K., Arora Y., Kumar R., Mehta V.S., Singh P., Maroon J.C., Bansal R., Sandal K., Roy R.G., Samkaria A., Sharma S., Sandhilya S., Gaur S., Parvathi S., Joshi M. (2023). Glutathione depletion and concomitant elevation of susceptibility in patients with Parkinson’s Disease: State-of-the-art MR spectroscopy and neuropsychological study.. ACS Chem. Neurosci..

[40] Bharath S., Hsu M., Kaur D., Rajagopalan S., Andersen J.K. (2002). Glutathione, iron and Parkinson’s disease.. Biochem. Pharmacol..

[41] Wang T., Liu W., Zhang Q., Jiao J., Wang Z., Gao G., Yang H. (2024). 4-oxo-2-nonenal- and agitation-induced aggregates of α-synuclein and phosphorylated α-synuclein with distinct biophysical properties and biomedical applications.. Cells.

[42] Lin X.M., Pan M.H., Sun J., Wang M., Huang Z.H., Wang G., Wang R., Gong H.B., Huang R.T., Huang F., Sun W.Y., Liu H.Z., Kurihara H., Li Y.F., Duan W.J., He R.R. (2023). Membrane phospholipid peroxidation promotes loss of dopaminergic neurons in psychological stress‐induced Parkinson’s disease susceptibility.. Aging Cell.

[43] Cai Y., Lendel C., Österlund L., Kasrayan A., Lannfelt L., Ingelsson M., Nikolajeff F., Karlsson M., Bergström J. (2015). Changes in secondary structure of α-synuclein during oligomerization induced by reactive aldehydes.. Biochem. Biophys. Res. Commun..

[44] Almandoz-Gil L., Welander H., Ihse E., Khoonsari P.E., Musunuri S., Lendel C., Sigvardson J., Karlsson M., Ingelsson M., Kultima K., Bergström J. (2017). Low molar excess of 4-oxo-2-nonenal and 4-hydroxy-2-nonenal promote oligomerization of alpha-synuclein through different pathways.. Free Radic. Biol. Med..

[45] Näsström T., Fagerqvist T., Barbu M., Karlsson M., Nikolajeff F., Kasrayan A., Ekberg M., Lannfelt L., Ingelsson M., Bergström J. (2011). The lipid peroxidation products 4-oxo-2-nonenal and 4-hydroxy-2-nonenal promote the formation of α-synuclein oligomers with distinct biochemical, morphological, and functional properties.. Free Radic. Biol. Med..

[46] Acosta G., Race N., Herr S., Fernandez J., Tang J., Rogers E., Shi R. (2019). Acrolein-mediated alpha-synuclein pathology involvement in the early post-injury pathogenesis of mild blast-induced Parkinsonian neurodegeneration.. Mol. Cell. Neurosci..

[47] Wang Y.T., Lin H.C., Zhao W.Z., Huang H.J., Lo Y.L., Wang H.T., Lin A.M.Y. (2017). Acrolein acts as a neurotoxin in the nigrostriatal dopaminergic system of rat: Involvement of α-synuclein aggregation and programmed cell death.. Sci. Rep..

[48] Dexter D.T., Carter C.J., Wells F.R., Javoy-Agid F., Agid Y., Lees A., Jenner P., Marsden C.D. (1989). Basal lipid peroxidation in substantia nigra is increased in Parkinson’s disease.. J. Neurochem..

[49] Jenner P., Dexter D.T., Sian J., Schapira A.H., Marsden C.D. (1992). Oxidative stress as a cause of nigral cell death in Parkinson’s disease and incidental Lewy body disease. The royal kings and queens Parkinson’s disease research group.. Ann. Neurol..

[50] Farooqui T., Farooqui A.A. (2011). Lipid-mediated oxidative stress and inflammation in the pathogenesis of Parkinson’s disease.. Parkinsons Dis..

[51] Yoritaka A., Hattori N., Uchida K., Tanaka M., Stadtman E.R., Mizuno Y. (1996). Immunohistochemical detection of 4-hydroxynonenal protein adducts in Parkinson disease.. Proc. Natl. Acad. Sci. USA.

[52] Castellani R.J., Perry G., Siedlak S.L., Nunomura A., Shimohama S., Zhang J., Montine T., Sayre L.M., Smith M.A. (2002). Hydroxynonenal adducts indicate a role for lipid peroxidation in neocortical and brainstem Lewy bodies in humans.. Neurosci. Lett..

[53] Monroy C.A., Doorn J.A., Roman D.L. (2013). Modification and functional inhibition of regulator of G-protein signaling 4 (RGS4) by 4-hydroxy-2-nonenal.. Chem. Res. Toxicol..

[54] Shi L., Lin Y., Jiao Y., Herr S.A., Tang J., Rogers E., Chen Z., Shi R. (2021). Acrolein scavenger dimercaprol offers neuroprotection in an animal model of Parkinson’s disease: Implication of acrolein and TRPA1.. Transl. Neurodegener..

[55] van der Burg J.M.M., Björkqvist M., Brundin P. (2009). Beyond the brain: Widespread pathology in Huntington’s disease.. Lancet Neurol..

[56] Shafie A., Ashour A.A., Anwar S., Anjum F., Hassan M.I. (2024). Exploring molecular mechanisms, therapeutic strategies, and clinical manifestations of Huntington’s disease.. Arch. Pharm. Res..

[57] Oosterloo M., Touze A., Byrne L.M., Achenbach J., Aksoy H., Coleman A., Lammert D., Nance M., Nopoulos P., Reilmann R., Saft C., Santini H., Squitieri F., Tabrizi S., Burgunder J.M., Quarrell O. (2024). Pediatric Huntington disease working group of the European Huntington disease network. Clinical review of Juvenile Huntington’s disease.. J. Huntingtons Dis..

[58] Wells R.G., Neilson L.E., McHill A.W., Hiller A.L. (2024). Dietary fasting and time-restricted eating in Huntington’s disease: therapeutic potential and underlying mechanisms.. Transl. Neurodegener..

[59] Brondani M., Roginski A.C., Ribeiro R.T., de Medeiros M.P., Hoffmann C.I.H., Wajner M., Leipnitz G., Seminotti B. (2023). Mitochondrial dysfunction, oxidative stress, ER stress and mitochondria-ER crosstalk alterations in a chemical rat model of Huntington’s disease: Potential benefits of bezafibrate.. Toxicol. Lett..

[60] Hariharan A., Shetty S., Shirole T., Jagtap A.G. (2014). Potential of protease inhibitor in 3-nitropropionic acid induced Huntington’s disease like symptoms: Mitochondrial dysfunction and neurodegeneration.. Neurotoxicology.

[61] Maity S., Komal P., Kumar V., Saxena A., Tungekar A., Chandrasekar V. (2022). Impact of ER stress and er-mitochondrial crosstalk in Huntington’s disease.. Int. J. Mol. Sci..

[62] Browne S.E., Beal M.F. (2006). Oxidative damage in Huntington’s disease pathogenesis.. Antioxid. Redox Signal..

[63] Browne S.E., Mitochondria E. (2008). Mitochondria and Huntington’s disease pathogenesis: Insight from genetic and chemical models.. Ann. N. Y. Acad. Sci..

[64] Romano A., Serviddio G., Calcagnini S., Villani R., Giudetti A.M., Cassano T., Gaetani S. (2017). Linking lipid peroxidation and neuropsychiatric disorders: Focus on 4-hydroxy-2-nonenal.. Free Radic. Biol. Med..

[65] Fotoohi A., Moloudi M.R., Hosseini S., Hassanzadeh K., Feligioni M., Izadpanah E. (2021). A novel pharmacological protective role for safranal in an animal model of Huntington’s disease.. Neurochem. Res..

[66] Verma M.K., Goel R., Nandakumar K., Nemmani K.V.S. (2018). Bilateral quinolinic acid-induced lipid peroxidation, decreased striatal monoamine levels and neurobehavioral deficits are ameliorated by GIP receptor agonist D-Ala2 GIP in rat model of Huntington’s disease.. Eur. J. Pharmacol..

[67] Johri A., Beal M.F. (2012). Antioxidants in Huntington’s disease.. Biochim. Biophys. Acta Mol. Basis Dis..

[68] Alharbi K.S. (2024). Europinidin mitigates 3-NPA-induced Huntington’s disease symptoms in rats: A comprehensive analysis of oxidative stress, mitochondrial enzyme complex activity, pro-inflammatory markers and neurotransmitter alterations.. Biomedicines.

[69] D’Egidio F., Castelli V., Cimini A., d’Angelo M. (2023). Cell rearrangement and oxidant/antioxidant imbalance in Huntington’s disease.. Antioxidants.

[70] Lee J., Kosaras B., Del Signore S.J., Cormier K., McKee A., Ratan R.R., Kowall N.W., Ryu H. (2011). Modulation of lipid peroxidation and mitochondrial function improves neuropathology in Huntington’s disease mice.. Acta Neuropathol..

[71] Tang Q., Liu H., Shi X.J., Cheng Y. (2020). Blood oxidative stress marker aberrations in patients with Huntington’s disease: A meta-analysis study.. Oxid. Med. Cell. Longev..

[72] Chen L., Qin Y., Guo T., Zhu W., Lin J., Xing T., Duan X., Zhang Y., Ruan E., Li X., Yin P., Li S., Li X.J., Yang S. (2024). HAP40 modulates mutant Huntingtin aggregation and toxicity in Huntington’s disease mice.. Cell Death Dis..

[73] Saudou F., Humbert S. (2016). The biology of Huntingtin.. Neuron.

[74] Davranche A., Aviolat H., Zeder-Lutz G., Busso D., Altschuh D., Trottier Y., Klein F.A.C. (2011). Huntingtin affinity for partners is not changed by polyglutamine length: Aggregation itself triggers aberrant interactions.. Hum. Mol. Genet..

[75] Chen Y., Al-Nusaif M., Li S., Tan X., Yang H., Cai H., Le W. (2024). Progress on early diagnosing Alzheimer’s disease.. Front. Med..

[76] Hu S., Yang C., Luo H. (2022). Current trends in blood biomarker detection and imaging for Alzheimer’s disease.. Biosens. Bioelectron..

[77] Mi J., Liu C., Chen H., Qian Y., Zhu J., Zhang Y., Liang Y., Wang L., Ta D. (2024). Light on Alzheimer’s disease: From basic insights to preclinical studies.. Front. Aging Neurosci..

[78] Huang W.J., Zhang X., Chen W.W. (2016). Role of oxidative stress in Alzheimer’s disease.. Biomed. Rep..

[79] Sultana R., Ravagna A., Mohmmad-Abdul H., Calabrese V., Butterfield D.A. (2005). Ferulic acid ethyl ester protects neurons against amyloid β‐peptide(1-42)‐induced oxidative stress and neurotoxicity: Relationship to antioxidant activity.. J. Neurochem..

[80] Yu N., Pasha M., Chua J.J.E. (2024). Redox changes and cellular senescence in Alzheimer’s disease.. Redox Biol..

[81] Mark R.J., Lovell M.A., Markesbery W.R., Uchida K., Mattson M.P. (1997). A role for 4-hydroxynonenal, an aldehydic product of lipid peroxidation, in disruption of ion homeostasis and neuronal death induced by amyloid beta-peptide.. J. Neurochem..

[82] Yamashima T., Ota T., Mizukoshi E., Nakamura H., Yamamoto Y., Kikuchi M., Yamashita T., Kaneko S. (2020). Intake of ω-6 polyunsaturated fatty acid-rich vegetable oils and risk of lifestyle diseases.. Adv. Nutr..

[83] Yamashima T., Seike T., Oikawa S., Kobayashi H., Kido H., Yanagi M., Yamamiya D., Li S., Boontem P., Mizukoshi E. (2023). Hsp70.1 carbonylation induces lysosomal cell death for lifestyle-related diseases.. Front. Mol. Biosci..

[84] Yamashima T., Seike T., Mochly-Rosen D., Chen C.H., Kikuchi M., Mizukoshi E. (2023). Implication of the cooking oil-peroxidation product “hydroxynonenal” for Alzheimer’s disease.. Front. Aging Neurosci..

[85] Reed T.T., Pierce W.M., Markesbery W.R., Butterfield D.A. (2009). Proteomic identification of HNE-bound proteins in early Alzheimer disease: Insights into the role of lipid peroxidation in the progression of AD.. Brain Res..

[86] Williams T.I., Lynn B.C., Markesbery W.R., Lovell M.A. (2006). Increased levels of 4-hydroxynonenal and acrolein, neurotoxic markers of lipid peroxidation, in the brain in mild cognitive impairment and early Alzheimer’s disease.. Neurobiol. Aging.

[87] Singh M., Nam D.T., Arseneault M., Ramassamy C. (2010). Role of by-products of lipid oxidation in Alzheimer’s disease brain: A focus on acrolein.. J. Alzheimers Dis..

[88] Butterfield D.A., Sultana R. (2011). Methionine-35 of aβ(1-42): Importance for oxidative stress in Alzheimer disease.. J. Amino Acids.

[89] Boonpraman N., Yi S.S. (2024). NADPH oxidase 4 (NOX4) as a biomarker and therapeutic target in neurodegenerative diseases.. Neural Regen. Res..

[90] Boonpraman N., Yoon S., Kim C.Y., Moon J.S., Yi S.S. (2023). NOX4 as a critical effector mediating neuroinflammatory cytokines, myeloperoxidase and osteopontin, specifically in astrocytes in the hippocampus in Parkinson’s disease.. Redox Biol..

[91] Luengo E., Trigo-Alonso P., Fernández-Mendívil C., Nuñez Á., Campo M., Porrero C., García-Magro N., Negredo P., Senar S., Sánchez-Ramos C., Bernal J.A., Rábano A., Hoozemans J., Casas A.I., Schmidt H.H.H.W., López M.G. (2022). Implication of type 4 NADPH oxidase (NOX4) in tauopathy.. Redox Biol..

[92] Park M.W., Cha H.W., Kim J., Kim J.H., Yang H., Yoon S., Boonpraman N., Yi S.S., Yoo I.D., Moon J.S. (2021). NOX4 promotes ferroptosis of astrocytes by oxidative stress-induced lipid peroxidation *via* the impairment of mitochondrial metabolism in Alzheimer’s diseases.. Redox Biol..

[93] Renuka Sanotra M., Huang W.C., Silver S., Lin C.Y., Chang T.C., Nguyen D.P.Q., Lee C.K., Kao S.H., Chang-Cheng Shieh J., Lin Y.F. (2022). Serum levels of 4-hydroxynonenal adducts and responding autoantibodies correlate with the pathogenesis from hyperglycemia to Alzheimer’s disease.. Clin. Biochem..

[94] Sanotra M.R., Kao S.H., Lee C.K., Hsu C.H., Huang W.C., Chang T.C., Tu F.Y., Hsu I.U., Lin Y.F. (2023). Acrolein adducts and responding autoantibodies correlate with metabolic disturbance in Alzheimer’s disease.. Alzheimers Res. Ther..

[95] Fukuda M., Kanou F., Shimada N., Sawabe M., Saito Y., Murayama S., Hashimoto M., Maruyama N., Ishigami A. (2009). Elevated levels of 4-hydroxynonenal-histidine Michael adduct in the hippocampi of patients with Alzheimer’s disease.. Biomed. Res..

[96] Wei Y., Zhong S., Yang H., Wang X., Lv B., Bian Y., Pei Y., Xu C., Zhao Q., Wu Y., Luo D., Wang F., Sun H., Chen Y. (2024). Current therapy in amyotrophic lateral sclerosis (ALS): A review on past and future therapeutic strategies.. Eur. J. Med. Chem..

[97] Genge A., Wainwright S., Vande Velde C. (2024). Amyotrophic lateral sclerosis: Exploring pathophysiology in the context of treatment.. Amyotroph. Lateral Scler. Frontotemporal Degener..

[98] Hemerková P., Vališ M. (2021). Role of oxidative stress in the pathogenesis of amyotrophic lateral sclerosis: Antioxidant metalloenzymes and therapeutic strategies.. Biomolecules.

[99] Wang X.X., Chen W.Z., Li C., Xu R.S. (2024). Current potential pathogenic mechanisms of copper-zinc superoxide dismutase 1 (SOD1) in amyotrophic lateral sclerosis.. Rev. Neurosci..

[100] Barber S.C., Shaw P.J. (2010). Oxidative stress in ALS: Key role in motor neuron injury and therapeutic target.. Free Radic. Biol. Med..

[101] Carrera-Juliá S., Moreno M.L., Barrios C., de la Rubia Ortí J.E., Drehmer E. (2020). Antioxidant alternatives in the treatment of amyotrophic lateral sclerosis: A comprehensive review.. Front. Physiol..

[102] Carrera-Juliá S., Estrela J.M., Zacarés M., Navarro M.Á., Vega-Bello M.J., de la Rubia Ortí J.E., Moreno M.L., Drehmer E. (2023). Effect of the Mediterranean diet supplemented with nicotinamide riboside and pterostilbene and/or coconut oil on anthropometric variables in amyotrophic lateral sclerosis. A pilot study.. Front. Nutr..

[103] Carrera-Juliá S., Estrela J.M., Zacarés M., Navarro M.Á., Vega-Bello M.J., de la Rubia Ortí J.E., Moreno M.L., Drehmer E. (2024). Nutritional, clinical and sociodemographic profiles of spanish patients with amyotrophic lateral sclerosis.. Nutrients.

[104] Shichiri M. (2014). The role of lipid peroxidation in neurological disorders.. J. Clin. Biochem. Nutr..

[105] Schmitt F., Hussain G., Dupuis L., Loeffler J.P., Henriques A. (2014). A plural role for lipids in motor neuron diseases: Energy, signaling and structure.. Front. Cell. Neurosci..

[106] Pedersen W.A., Fu W., Keller J.N., Markesbery W.R., Appel S., Smith R.G., Kasarskis E., Mattson M.P. (1998). Protein modification by the lipid peroxidation product 4‐hydroxynonenal in the spinal cords of amyotrophic lateral sclerosis patients.. Ann. Neurol..

[107] Perluigi M., Fai Poon H., Hensley K., Pierce W.M., Klein J.B., Calabrese V., De Marco C., Butterfield D.A. (2005). Proteomic analysis of 4-hydroxy-2-nonenal-modified proteins in G93A-SOD1 transgenic mice-A model of familial amyotrophic lateral sclerosis.. Free Radic. Biol. Med..

[108] Smith R.G., Henry Y.K., Mattson M.P., Appel S.H. (1998). Presence of 4‐hydroxynonenal in cerebrospinal fluid of patients with sporadic amyotrophic lateral sclerosis.. Ann. Neurol..

[109] Chaves-Filho A.B., Pinto I.F.D., Dantas L.S., Xavier A.M., Inague A., Faria R.L., Medeiros M.H.G., Glezer I., Yoshinaga M.Y., Miyamoto S. (2019). Alterations in lipid metabolism of spinal cord linked to amyotrophic lateral sclerosis.. Sci. Rep..

[110] Zhai J., Ström A.L., Kilty R., Venkatakrishnan P., White J., Everson W.V., Smart E.J., Zhu H. (2009). Proteomic characterization of lipid raft proteins in amyotrophic lateral sclerosis mouse spinal cord.. FEBS J..

[111] (2020). FernÁndez-Eulate, G.; Ruiz-Sanz, J.I.; Riancho, J.; ZufirÍa, M.; GereÑu, G.; FernÁndez-TorrÓn, R.; Poza-Aldea, J.J.; Ondaro, J.; Espinal, J.B.; GonzÁlez-ChinchÓn, G.; Zulaica, M.; Ruiz-Larrea, M.B.; LÓpez De Munain, A.; Gil-Bea, F.J. A comprehensive serum lipidome profiling of amyotrophic lateral sclerosis.. Amyotroph. Lateral Scler. Frontotemporal Degener..

[112] Phan K., He Y., Bhatia S., Pickford R., McDonald G., Mazumder S., Timmins H.C., Hodges J.R., Piguet O., Dzamko N., Halliday G.M., Kiernan M.C., Kim W.S. (2022). Multiple pathways of lipid dysregulation in amyotrophic lateral sclerosis.. Brain Commun..

[113] Ashizawa T., Xia G. (2016). Ataxia.. Continuum (Minneap. Minn.).

[114] Pandolfo M., Manto M. (2013). Cerebellar and afferent ataxias.. Continuum (Minneap. Minn.).

[115] Manto M., Marmolino D. (2009). Cerebellar ataxias.. Curr. Opin. Neurol..

[116] Rudaks L.I., Yeow D., Ng K., Deveson I.W., Kennerson M.L., Kumar K.R. (2024). An update on the adult-onset hereditary cerebellar ataxias: Novel genetic causes and new diagnostic approaches.. Cerebellum.

[117] Eisel M.L.S., Burns M., Ashizawa T., Byrne B., Corti M., Subramony S.H. (2024). Emerging therapies in hereditary ataxias.. Trends Mol. Med..

[118] Soto-Piña A.E., Pulido-Alvarado C.C., Dulski J., Wszolek Z.K., Magaña J.J. (2024). Specific biomarkers in spinocerebellar ataxia type 3: A systematic review of their potential uses in disease staging and treatment assessment.. Int. J. Mol. Sci..

[119] Pandolfo M. (2009). Friedreich ataxia: The clinical picture.. J. Neurol..

[120] Alqurashi R.M., Aladwani A., Alosaimi T.H., Yousef D.B., Alkhaldi M.H., Amer M.G. (2023). Awareness of the effect of vitamin B12 deficiency on the nervous system among the general population in Taif, Saudi Arabia.. Cureus.

[121] Omura Y., Ota K., Takasu A., Suzuki T. (2024). Vitamin B1 deficiency identified from incidental detection of Hyperlactatemia: A case report.. Medicina (Kaunas).

[122] Lee J.H. (2024). Targeting the ATM pathway in cancer: Opportunities, challenges and personalized therapeutic strategies.. Cancer Treat. Rev..

[123] Varadhan V., Manikandan M.S., Nagarajan A., Palaniyandi T., Ravi M., Sankareswaran S.K., Baskar G., Wahab M.R.A., Surendran H. (2024). Ataxia-Telangiectasia Mutated (ATM) gene signaling pathways in human cancers and their therapeutic implications.. Pathol. Res. Pract..

[124] Lee J.H. (2024). Oxidative stress and the multifaceted roles of ATM in maintaining cellular redox homeostasis.. Redox Biol..

[125] Lee J.H., Paull T.T. (2020). Mitochondria at the crossroads of ATM-mediated stress signaling and regulation of reactive oxygen species.. Redox Biol..

[126] Leeson H.C., Aguado J., Gómez-Inclán C., Chaggar H.K., Fard A.T., Hunter Z., Lavin M.F., Mackay-Sim A., Wolvetang E.J. (2024). Ataxia telangiectasia patient-derived neuronal and brain organoid models reveal mitochondrial dysfunction and oxidative stress.. Neurobiol. Dis..

[127] Sanz-Alcázar A., Britti E., Delaspre F., Medina-Carbonero M., Pazos-Gil M., Tamarit J., Ros J., Cabiscol E. (2024). Mitochondrial impairment, decreased sirtuin activity and protein acetylation in dorsal root ganglia in Friedreich Ataxia models.. Cell. Mol. Life Sci..

[128] Lynch D.R., Mathews K.D., Perlman S., Zesiewicz T., Subramony S., Omidvar O., Vogel A.P., Krtolica A., Litterman N., van der Ploeg L., Heerinckx F., Milner P., Midei M. (2023). Double blind trial of a deuterated form of linoleic acid (RT001) in Friedreich ataxia.. J. Neurol..

[129] La Rosa P., Petrillo S., Turchi R., Berardinelli F., Schirinzi T., Vasco G., Lettieri-Barbato D., Fiorenza M.T., Bertini E.S., Aquilano K., Piemonte F. (2021). The Nrf2 induction prevents ferroptosis in Friedreich’s Ataxia.. Redox Biol..

[130] Abeti R., Parkinson M.H., Hargreaves I.P., Angelova P.R., Sandi C., Pook M.A., Giunti P., Abramov A.Y. (2016). Mitochondrial energy imbalance and lipid peroxidation cause cell death in Friedreich’s ataxia.. Cell Death Dis..

[131] Abeti R., Uzun E., Renganathan I., Honda T., Pook M.A., Giunti P. (2015). Targeting lipid peroxidation and mitochondrial imbalance in Friedreich’s ataxia.. Pharmacol. Res..

[132] Barrera G., Pizzimenti S., Daga M., Dianzani C., Arcaro A., Cetrangolo G.P., Giordano G., Cucci M.A., Graf M., Gentile F. (2018). Lipid peroxidation-derived aldehydes, 4-hydroxynonenal and malondialdehyde in aging-related disorders.. Antioxidants.

[133] Leung T.C.S., Fields E., Rana N., Shen R.Y.L., Bernstein A.E., Cook A.A., Phillips D.E., Watt A.J. (2024). Mitochondrial damage and impaired mitophagy contribute to disease progression in SCA6.. Acta Neuropathol..

[134] Chen C., Merrill R.A., Jong C.J., Strack S. (2024). Driving mitochondrial fission improves cognitive, but not motor deficits in a mouse model of ataxia of charlevoix-saguenay.. Cerebellum.

[135] Chaudhary P., Sharma R., Sahu M., Vishwanatha J.K., Awasthi S., Awasthi Y.C. (2013). 4-Hydroxynonenal induces G2/M phase cell cycle arrest by activation of the ataxia telangiectasia mutated and Rad3-related protein (ATR)/checkpoint kinase 1 (Chk1) signaling pathway.. J. Biol. Chem..

[136] Blignaut M., Harries S., Lochner A., Huisamen B. (2021). Ataxia telangiectasia mutated protein kinase: A potential master puppeteer of oxidative stress-induced metabolic recycling.. Oxid. Med. Cell. Long..

[137] Maciejczyk M., Heropolitanska-Pliszka E., Pietrucha B., Sawicka-Powierza J., Bernatowska E., Wolska-Kusnierz B., Pac M., Car H., Zalewska A., Mikoluc B. (2019). Antioxidant defense, redox homeostasis, and oxidative damage in children with Ataxia telangiectasia and Nijmegen breakage syndrome.. Front. Immunol..

[138] Andrade I.G.A., Suano-Souza F.I., Fonseca F.L.A., Lago C.S.A., Sarni R.O.S. (2021). Selenium levels and glutathione peroxidase activity in patients with ataxia-telangiectasia: Association with oxidative stress and lipid status biomarkers.. Orphanet J. Rare Dis..

[139] Zhang S., Eitan E., Wu T.Y., Mattson M.P. (2018). Intercellular transfer of pathogenic α-synuclein by extracellular vesicles is induced by the lipid peroxidation product 4-hydroxynonenal.. Neurobiol. Aging.

[140] Bae E.J., Ho D.H., Park E., Jung J.W., Cho K., Hong J.H., Lee H.J., Kim K.P., Lee S.J. (2013). Lipid peroxidation product 4-hydroxy-2-nonenal promotes seeding-capable oligomer formation and cell-to-cell transfer of α-synuclein.. Antioxid. Redox Signal..

[141] Qin Z., Hu D., Han S., Reaney S.H., Di Monte D.A., Fink A.L. (2007). Effect of 4-hydroxy-2-nonenal modification on alpha-synuclein aggregation.. J. Biol. Chem..

[142] Deas E., Cremades N., Angelova P.R., Ludtmann M.H.R., Yao Z., Chen S., Horrocks M.H., Banushi B., Little D., Devine M.J., Gissen P., Klenerman D., Dobson C.M., Wood N.W., Gandhi S., Abramov A.Y. (2016). Alpha-synuclein oligomers interact with metal ions to induce oxidative stress and neuronal death in Parkinson’s disease.. Antioxid. Redox Signal..

[143] Tabner B.J., Turnbull S., El-Agnaf O., Allsop D. (2001). Production of reactive oxygen species from aggregating proteins implicated in Alzheimer’s disease, Parkinson’s disease and other neurodegenerative diseases.. Curr. Top. Med. Chem..

[144] Di Maio R., Barrett P.J., Hoffman E.K., Barrett C.W., Zharikov A., Borah A., Hu X., McCoy J., Chu C.T., Burton E.A., Hastings T.G., Greenamyre J.T. (2016). α-synuclein binds to TOM20 and inhibits mitochondrial protein import in Parkinson’s disease.. Sci. Transl. Med..

[145] Rani P., Krishnan S., Rani Cathrine C. (2017). Study on analysis of peripheral biomarkers for Alzheimer’s disease diagnosis.. Front. Neurol..

[146] Markesbery W.R., Lovell M.A. (1998). Four-hydroxynonenal, a product of lipid peroxidation, is increased in the brain in Alzheimer’s disease.. Neurobiol. Aging.

[147] Edzeamey F.J., Ramchunder Z., Pourzand C., Anjomani Virmouni S. (2024). Emerging antioxidant therapies in Friedreich’s ataxia.. Front. Pharmacol..

[148] Lana J.V., Rios A., Takeyama R., Santos N., Pires L., Santos G.S., Rodrigues I.J., Jeyaraman M., Purita J., Lana J.F. (2024). Nebulized glutathione as a key antioxidant for the treatment of oxidative stress in neurodegenerative conditions.. Nutrients.

[149] Upadhayay S., Kumar P. (2024). Mitochondrial targeted antioxidants as potential therapy for Huntington’s disease.. Pharmacol. Rep..

[150] Pei J., Palanisamy C.P., Natarajan P.M., Umapathy V.R., Roy J.R., Srinivasan G.P., Panagal M., Jayaraman S. (2024). Curcumin-loaded polymeric nanomaterials as a novel therapeutic strategy for Alzheimer’s disease: A comprehensive review.. Ageing Res. Rev..

[151] Shea M.K., Xuan A.Y., Booth S.L., Vitamin D., Vitamin D. (2024). Alzheimer’s disease and related dementia.. Adv. Food Nutr. Res..

[152] Kumar R.R., Singh L., Thakur A., Singh S., Kumar B. (2022). Role of vitamins in neurodegenerative diseases: A review.. CNS Neurol. Disord. Drug Targets.

[153] Icer M.A., Arslan N., Gezmen-Karadag M. (2021). Effects of vitamin E on neurodegenerative diseases: An update.. Acta Neurobiol. Exp. (Warsz.).

[154] Martinelli C., Pucci C., Battaglini M., Marino A., Ciofani G. (2020). Antioxidants and nanotechnology: Promises and limits of potentially disruptive approaches in the treatment of central nervous system diseases.. Adv. Healthc. Mater..

